# Medical students’ attitudes toward sleeping pill usage: A cross-sectional study

**DOI:** 10.3389/fpsyt.2022.1007141

**Published:** 2022-12-20

**Authors:** Moudi M. Alasmari, Raghad S. Alkanani, Asmaa S. Alshareef, Shahd S. Alsulmi, Reuof I. Althegfi, Teef A. Bokhari, Mona Y. Alsheikh, Heba K. Alshaeri

**Affiliations:** ^1^College of Medicine, King Saud bin Abdulaziz University for Health Sciences, Jeddah, Saudi Arabia; ^2^King Abdullah International Medical Research Center (KAIMRC), Jeddah, Saudi Arabia; ^3^Department of Clinical Pharmacy, College of Pharmacy, Taif University, Taif, Saudi Arabia; ^4^Department of Pharmaceutical Sciences, Fakeeh College for Medical Sciences, Jeddah, Saudi Arabia

**Keywords:** sleeping pills, medical students, misuse, self-prescription, attitude, prevalence

## Abstract

Medical students experience significant stress, which may interfere with their sleeping routines; consequently, they are at a higher risk of misusing sleeping pills. Therefore, this cross-sectional convenience sampling study aims to evaluate medical students’ attitudes toward the usage of sleeping pills, as well as the prevalence of their misuse and the associated factors. A self-administered online questionnaire survey was completed by 338 medical students at the College of Medicine of the King Saud bin Abdulaziz University for Health Sciences. Of 338 participants, 84 (24.85%) reported using sleeping pills. The prevalence of sleeping pill misuse was 26.63% (*n* = 90). The most common misuse behavior of sleeping pills was self-prescription (*n* = 72, 21.30%). The factors associated with sleeping pill misuse were stimulants usage (*n* = 69, 92%, *p* = 0.0072), high grade point average (*n* = 63, 84%, *p* = 0.046), preclinical years (*n* = 52, 69.3%, *p* = 0.042), and longer average time required to fall asleep (*n* = 53, 70.7%, *p* = 0.008). The prevalence of sleeping pill misuse is high among medical students, particularly among those in preclinical years. Therefore, enhancing awareness regarding sleeping pill misuse is crucial. This can be achieved through campaigns, workshops, and providing information regarding the dangers of sleeping pill misuse in the curriculum.

## 1 Introduction

Medical students are often vulnerable to anxiety and stress due to medicine being a highly demanding career choice, both academically and professionally. Excessive stress can result in health problems, which can lower students’ self-esteem and affect their academic performance. Medical students may experience harmful effects as a consequence of excessive stress, which could impact their cognitive functioning and learning. If left unmanaged, any form of stress can lead to sleeping problems, burnout, and dropout (1–4).

Research from Canada and the United States indicates a higher prevalence of depression, anxiety, and psychological issues among medical students, compared with the general population (5). Possible sources of recurrent stress and anxiety include high curriculum difficulty, which requires students to cover large amounts of information within a short time span, and frequent examinations. Other sources of stress involve personal competency, endurance, and external life events. Furthermore, an increase in concern was linked to a rise in stress and anxiety. Meanwhile, social and personal issues in students’ daily lives comprise another source of stress and anxiety that must be dealt with through adequate stress management strategies (6, 7).

Among Saudi students of the King Faisal University’s College of Medicine (COM), the prevalence of stress was reported to be 53% in 2013 (8). Moreover, research conducted at a medical university in the United Arab Emirates revealed a significant association between stress and psychological morbidity (9). Another study conducted at the King Saud bin Abdulaziz University for Health Sciences revealed a significant link between poor sleep quality and stress (1). Students who experienced stress were more likely to have poor sleep quality, while students with a cumulative grade point average (GPA) lower than 4.25 were approximately four times more likely to have poor sleep quality (1). Psychological stress can result in depression and have other negative effects on students, such as reduced performance in classes or clinical practice and poor sleep quality (8, 10, 11). Stress is a major problem for medical students both in Saudi Arabia and worldwide. Therefore, investigating stress management techniques for this population is crucial. Some effective coping strategies for stress include time management and academic counseling (12). However, some students may indulge in unhealthy coping behaviors, such as the misuse of drugs.

According to a study conducted at a Pakistani medical school, drug misuse was reported by 4.9% of students (13). Moreover, studies have shown that the most common motives for prescription drug misuse among university students include personal enhancement related to sports and educational attainment, mental health (e.g., sleep disturbances and anxiety), or physical health (e.g., managing pre-existing illnesses). Fewer than half of the users said they were involved in prescription drug misuse for recreational purposes (e.g., to party, get high, experiment) (14).

The World Health Organization defines the misuse of medication as “the use of a substance for a purpose not consistent with legal or medical guidelines.” Additionally, self-prescription is defined as “the selection and use of medicines/medicinal products, including herbal and traditional products by individuals, to treat self-recognized illness or symptoms, or the intermittent or continued use of a medication prescribed by a physician for chronic or recurring diseases or symptoms” (15). A study conducted at King Khalid University revealed that 98.7% of students reported self-medication. The primary sources of information for self-medication were self-knowledge, relatives, pharmacists, and friends (16).

Medication misuse can negatively affect students’ performance as well as their psychological and physical health, and can cause adverse long-term consequences (17). One of the most commonly abused categories of prescription medications is Central Nervous System (CNS) depressant drugs; these medications slow brain activity, making them useful for treating anxiety, panic, acute stress reactions, and sleep disorders (18, 19). CNS depressants include sedatives, drugs used for treating sleep disorders, hypnotics, and sleeping pills (18). The term “sleeping pill” is a generic term referring to prescription or over-the-counter drugs that promote, induce, or maintain sleep (20). Sleeping pill use can have serious side effects and may even result in addiction if used over a long period, leading to long-term health consequences, including disturbed thinking, headaches, constipation or diarrhea, and muscle weakness (20).

In recent years, the use of CNS-depressant drugs by medical students and doctors has attracted attention and concern from researchers, educational institutions, and medical societies. A study conducted at the King Saud University in 2011 found that 17% of medical students reported sedative drug use at some time since enrollment (12). Other studies have shown similar findings, thus confirming that the use of CNS-depressant drugs is prevalent among medical students, which makes this a sensitive and important issue (21, 22).

Although previous studies indicate that medical students’ use of CNS-depressant drugs is prevalent, no study has assessed Saudi Arabian medical students’ attitudes toward sleeping pill usage or identified the factors associated with the misuse. Therefore, this study primarily aimed to investigate the attitudes of medical students at the King Saud bin Abdulaziz University for Health Sciences toward sleeping pill usage. We also aimed to estimate the prevalence of sleeping pill misuse and determine the factors that contribute to such behavior.

## 2 Materials and methods

### 2.1 Study design, setting, and participants

A self-administered online questionnaire survey was conducted in this cross-sectional convenience sampling study. The study included students attending the college of medicine (COM) at the King Saud bin Abdulaziz University for Health Sciences. The COM is located within the King Abdulaziz Medical City and has two branches—in Riyadh and in Jeddah. In the 2019–2020 academic year, there were 1,177 and 781 COM attendees at the Riyadh and Jeddah branches, respectively, totaling 1,958 students. The only inclusion criterion was regularly attending the COM; thus, no exclusion criteria were stipulated. The sample size was calculated using Raosoft software (23). The required sample size was estimated at a 95% confidence level with a 50% assumed prevalence of sleeping pill usage and a 5% margin of error. The recommended sample size was determined to be 322. The survey was distributed from 2 September 2021 to 14 December 2021. The survey questionnaire was developed by the authors, based on a previously published validated questionnaire, with minor modifications, to evaluate both quantitative and qualitative variables (12). To retest the questionnaire, a pilot study was performed on a sample of 10 students to assess data-gathering procedures and ensure the reliability of the questionnaire. Any required modifications were then made. The participants were informed of the purpose of the research and their right to refuse participation, and that their personal information would be kept completely confidential.

### 2.2 Data collection

The self-administered questionnaire comprised 20 questions, divided into three sections. The first section was aimed at collecting socio-demographic information: age, gender, residence, stream, college branch (Riyadh or Jeddah), academic year, GPA, and smoking status. The university accepts students who have graduated from other specialties; this path is called stream II, while stream I refers to students whose first specialty after high school was medicine. Academic years were also subdivided, based on the university’s curriculum, where the first year is a foundation year in which students are not enrolled in the COM; years 2–4 comprise the preclinical phase, and years 5 and 6 comprise the clinical phase. The first section of the survey also included questions regarding physical exercise, use of any type of stimulants during the exam period, and additional information regarding sleeping status to assess sleep pattern and quality and any sleeping disorders. The third section was preceded by a one-question section where participants were asked whether they had ever used sleeping pills after entering COM; based on their answers, respondents were either directed to the third section or to the end of the survey. The third section comprised four questions that investigated participants’ use of sleeping pills, wherein they specified the name of the drug used, either by writing the name or choosing from a list containing the names of some of the most commonly used sleeping pills. The students were also asked about the reasons for their sleeping pill usage, whether they consumed the pills in greater amounts than recommended, and their frequency of consumption. In summary, the third section was intended to gather information regarding the misuse of sleeping pills and to determine its prevalence.

### 2.3 Data analysis

Data analysis was conducted using Microsoft Excel 2016 and IBM^®^ SPSS Statistics version 28.0 software (IBM Corp., Armonk, NY, USA). Descriptive statistics were used, and the dataset (demographic characteristics, sleeping status, prevalence of sleeping disorders, sleeping pill usage, and attitudes) are presented as frequencies (*N*) and percentages (%). A chi-squared test and a Fisher’s exact test were performed to determine the association of demographic characteristics with medical students’ attitudes. A *p*-value of <0.05 was considered statistically significant.

### 2.4 Ethical consideration

The King Abdullah International Medical Research Center provided ethical approval for this study (IRB number: SP21J/025/02). All participants provided their informed consent, after we assured them of their anonymity and data confidentiality. The data were gathered anonymously and recorded in an Excel document on Google Forms. No information that may be used to identify a participant was sought.

## 3 Results

A total of 338 medical students responded to the online questionnaire. As illustrated in the summary of demographic characteristics (Table 1). Most respondents (*n* = 220, 65.1%) were aged 19–21 years, less than a third (*n* = 102, 30.2%) were 22–24 years, and only a few (*n* = 16, 4.7%) were older than 24 years. The sample included 141 (41.7%) male students and 197 (58.3%) female students. Almost all participants reported residing with their families (*n* = 324, 95.9%), while only a few reported living alone (*n* = 10, 3.0%) or with friends (*n* = 4, 1.2%). A higher proportion of respondents were stream I students (*n* = 316, 93.5%), and the remainder (*n* = 22, 6.5%) were stream II. Most of the students were from the Jeddah branch (*n* = 229, 67.8%), and the rest (*n* = 109, 32.2%) were from Riyadh. Further, 258 students (76.3%) were in pre-clinical years, and 80 (23.7%) in clinical years. Most of the respondents reported having a GPA of 4.5–5 (*n* = 282, 83.4%) and using stimulants (*n* = 271, 80.2%). Moreover, most respondents were non-smokers (*n* = 309, 91.4%). More than half of the respondents exercised regularly and had a night and day sleeping pattern. Less than one-third (*n* = 61, 18%) of the respondents reported poor sleep quality. Similarly, 18.9% of respondents (*n* = 64) reported requiring less than 10 min to fall asleep.

**TABLE 1:** Summary of participants’ demographic characteristics.

Baseline characteristics	*n*	%
**Age range, years**		
19–21	220	65.1
22–24	102	30.2
25–27	13	3.8
28–30	3	0.9
**Gender**		
Male	141	41.7
Female	197	58.3
**Residence**		
With family	324	95.9
With friends	4	1.2
Alone	10	3.0
**Stream**		
Stream I	316	93.5
Stream II	22	6.5
**College branch**		
Jeddah	229	67.8
Riyadh	109	32.2
**Academic year**		
Preclinical years (2–4)	258	76.4
Clinical years (5–6)	80	23.7
**Grade point average (GPA)**		
4.50–5.00	282	83.4
4.00–4.49	49	14.5
3.50–3.99	6	1.8
3.00–3.49	1	0.3
**Smoking**		
Yes	29	8.57
No	309	91.4
**Exercise**		
Yes	143	42.3
No	195	57.70
**Stimulant use**		
Yes	271	80.1
No	67	19.8
**Sleeping average, hours**		
<6	105	31.0
6–10	225	66.5
>10	8	2.36
**Falling asleep average, minutes**		
<10	64	18.9
10–20	140	41.4
>20	134	39.6
**Sleeping pattern**		
Night only	146	43.2
Night and day	192	56.8
**Sleep quality**		
Poor	61	18.0
Good	164	48.5
Excellent	113	33.4
Sleeping pill usage	84	24.9

**FIGURE 1:**
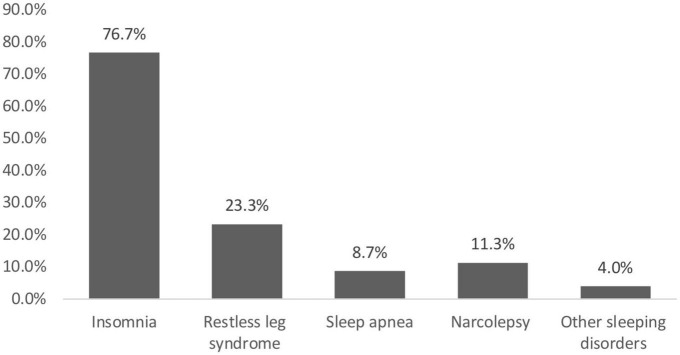
Prevalence of sleeping disorders.

Survey findings reveal that 150 (44.4%) students had sleeping disorders. As illustrated in Figure 1, 115 (76.7%) of them suffered from insomnia, 35 (23.3%) had restless leg syndrome, 17 (11.3%) had narcolepsy, 13 (8.7%) experienced sleep apnea, and 6 (4%) had other sleeping disorders, which including waking up frequently, excessive daytime sleepiness, allergic rhinosinusitis and postnasal drip, nightmares, and one student reported waking up tired no matter how long he slept.

**FIGURE 2:**
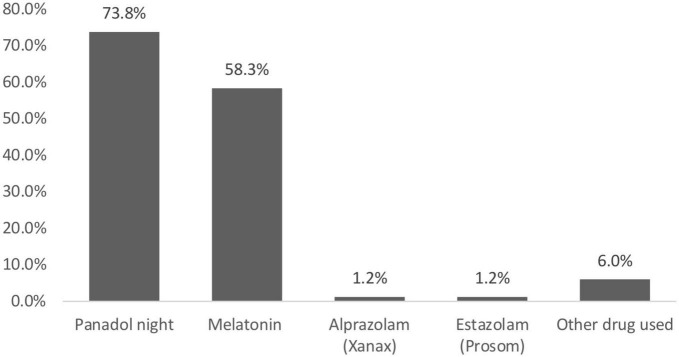
Medical students’ attitude toward sleeping pill usage.

Attitudes toward the use of sleeping pills were assessed for students who reported using sleeping pills (*n* = 84, 24.9%). Of the 84 respondents, only 11 (13.1%) reported using sleeping pills with a doctor’s prescription. Conversely, more than two-thirds (*n* = 72, 85.7%) of students self-prescribed the medication, while only one (1.2%) respondent reported using sleeping pills for recreational purposes. In general, almost all respondents used sleeping pills in moderate amounts (*n* = 74, 88.1%), and only 10 (11.9%) reported usage in higher amounts. Frequency analysis of sleeping pill usage showed that only 6 (7.1%) respondents used sleeping pills almost always; similarly, 6 (7.1%) respondents used sleeping pills after short or long holidays. In addition, 36 (42.9%) respondents had used sleeping pills only once, and 31 (36.9%) used them on separate occasions (Figure 2).

**FIGURE 3:**
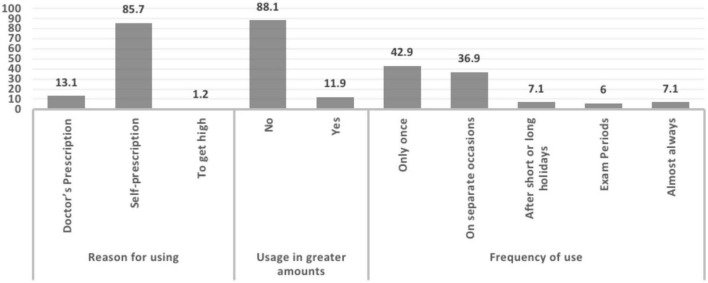
Prevalence of sleeping pill usage.

The most commonly used sleeping pill was Panadol Night (*n* = 62, 73.8%), followed by melatonin pills (*n* = 49, 58.3%). Alprazolam (Xanax) and estazolam (Prosom) had the same prevalence (1.2%). Some students reported using other sleeping pills (6%), namely, pseudoephedrine and lorazepam pills (Figure 3).

Of the 84 respondents who reported using sleeping pills, 75 (89.3%) students misused the pills. An analysis showed that academic year (*p* = 0.042), GPA (*p* = 0.046), stimulants use (*p* = 0.007), and longer average time required to fall asleep (*p* = 0.008) were significantly associated with sleeping pill misuse. Out of 197 female participated in this study (24.9%) *n* = 49 reported misusing of sleeping pills. Additionally, in 141 male only (18.4%) *n* = 21 misused sleeping pills. In 324 students who live with their family (21.6%) *n* = 70 misused sleeping pills, while (50%) *n* = 2 out of 4 students living with friends reported misusing sleeping pills. In addition, (30%) *n* = 3 out of 10 who live alone, reported misusing sleeping pills. Moreover, 52 (20.2%) students of preclinical years misused sleeping pills compared to 23 (28.75%) students of clinical years. Among students who had a high GPA, 63 (22.3%) students misused sleeping pills while respondents with a lower GPA who misused sleeping pills were 12 (24.5%) students. Among 29 students who are smokers (20.7%) *n* = 6 reported misusing sleeping pills; however, (22.3%) *n* = 69 of who aren’t smokers reported misusing sleeping pills. (21.7%) *n* = 31 of 143 who exercise, reported misusing sleeping pills, while (22,6%) *n* = 44 of who doesn’t exercise reported misusing sleeping pills. Out of 271 students who reported stimulants use, (25.5%) *n* = 69 reported misusing sleeping pills. However, (9.0%) *n* = 6 of students who doesn’t use stimulants, reported misusing sleeping pills. Among students who reported less than 6 h of sleep, 26 (24.8%) students were found to misuse sleeping pills; in contrast, 1 (12.5%) student who reported more than 10 h of sleep misused sleeping pills. While 53 (39.6%) respondents who needed more than 20 min, on average, to fall asleep reported misuse of sleeping pills, 4 (6.25%) respondents who needed less than 10 min to fall asleep misused sleeping pills. Moreover, 48 (25.0%) respondents who reported sleeping at night and day misused sleeping pills while 27 (18.5%) respondents who sleep at night only reported sleeping pills misuse. There were 61 students with a poor sleep quality, only (24.6%) *n* = 15 reported misusing sleeping pills; Moreover, out of 164 students with good sleeping quality, (24.4%) *n* = 40 reported misusing sleeping pills. In 113 students with excellent sleeping quality, (17.7%) *n* = 20 reported sleeping pill misuse (Table 2).

**TABLE 2:** Factors associated with sleeping pill misuse.

Factor		Sleeping pill misuse		Total	*P*-value
		Yes	No		
Gender					0.611
Male	*n*	26	115	141	
	%	18.4	81.6	100.0	
Female	*n*	49	148	197	
	%	24.9	75.1	100.0	
Residence					0.270
With family	*n*	70	254	324	
	%	21.6	78.4	100.0	
With friends	*n*	2	2	4	
	%	50.0	50.0	100.0	
Alone	*n*	3	7	10	
	%	30.0	70.0	100.0	
Academic year					0.042
Preclinical (2–4)	*n*	52	206	258	
	%	20.2	79.8	100.0	
Clinical (5–6)	*n*	23	57	80	
	%	28.75	71.25	100.00	
Grade point average (GPA)					0.046
4.50–5.00	*n*	63	219	282	
	%	22.3	77.7	100.0	
4.00–4.49	*n*	12	37	49	
	%	24.5	75.5	100.0	
Smoking					0.695
Yes	*n*	6	23	29	
	%	20.7	79.3	100.0	
No	*n*	69	240	309	
	%	22.3	77.7	100.0	
Exercise					0.081
Yes	*n*	31	112	143	
	%	21.7	78.3	100.0	
No	*n*	44	151	195	
	%	22.6	77.4	100.0	
Stimulants use					0.007
Yes	*n*	69	202	271	
	%	25.5	74.5	100.0	
No	*n*	6	61	67	
	%	9.0	91.0	100.0	
Sleeping average, hours					0.250
<6	*n*	26	79	105	
	%	24.8	75.2	100.0	
6–10	*n*	48	177	225	
	%	21.3	78.7	100.0	
>10	*n*	1	7	8	
	%	12.5	87.5	100.0	
Falling asleep average, minutes					0.008
<10	*n*	4	60	64	
	%	6.25	93.75	100.00	
10–20	*n*	18	122	140	
	%	12.9	87.1	100.0	
>20	*n*	53	81	134	
	%	39.6	60.4	100.0	
Sleeping pattern					0.933
Night only	*n*	27	119	146	
	%	18.5	81.5	100.0	
Night and day	*n*	48	144	192	
	%	25.0	75.0	100.0	
Sleep quality					0.129
Poor	*n*	15	46	61	
	%	24.6	75.4	100.0	
Good	*n*	40	124	164	
	%	24.4	75.6	100.0	
Excellent	*n*	20	93	113	
	%	17.7	82.3	100.0	

## 4 Discussion

This study assessed medical students’ attitudes toward sleeping pill usage. The study also estimated the prevalence of sleeping pill misuse and the factors associated with it among Saudi medical students.

The misuse of sleeping pills can have negative consequences, affecting one’s mental and physical wellbeing. While limited international studies have reported similar findings, this discussion involves multiple national studies that have revealed findings consistent with those in our study. However, it should be noted that this cross-sectional study was conducted in 2021–2022; therefore, the COVID-19 pandemic may have potentially affected the findings.

Medical students’ attitudes toward the usage of sleeping pills were assessed after considering the following as indicators of misuse: use of sleeping pills almost daily, use in greater amounts than the recommended dose, self-prescription, and use for recreational purposes. Most students self-prescribed sleeping pills, which was considered a misuse attitude. Similar studies have shown self-prescription to be a common issue among medical students; self-prescription is exacerbated by easy accessibility and the availability of several types of over-the-counter drugs, such as Panadol Night and melatonin (24, 25). Although self-prescribed medications can have some benefits when used appropriately, it is a dangerous practice that can delay an accurate medical diagnosis and result in adverse effects, drug interactions, and developing dependence, thereby aggravating what was intended to be treated (26).

The analysis of respondents’ demographic variables revealed that several factors were significantly associated with sleeping pill misuse. Students who were in their preclinical years of college were found to misuse sleeping pills more frequently than those in their clinical years (*p* = 0.042). According to a study conducted in Brazil on sleep quality among medical students, students in the early years of medical school had poorer sleep quality, compared with those in later years (27). This can explain why the misuse of sleeping pills by students in their preclinical years was higher than that of those in their clinical years. Another variable that was significantly associated with sleeping pill misuse is GPA (*p* = 0.046). Students who had a higher GPA (4.50–5.00) were found to misuse sleeping pills more frequently than students with a lower GPA (4.00–4.49). However, a different study showed that students with lower GPA misused sedatives more frequently than those with a higher GPA. This inconsistency in findings may be due to the difference in medication class, tools, and population (12).

Almost all students who used sleeping pills were also found to be using stimulants (*p* = 0.007). Stimulant use can negatively impact the sleep-wake cycle, resulting in sleeping pill usage and misuse. It is well known that exercise enhances sleep quality (28). Interestingly, exercise was not associated with a decrease in sleeping pill misuse. This is consistent with the findings of a study conducted in India, which found no significant association between exercise and using sleeping pills (7).

An important variable to be mentioned is gender, although it was not statistically significant (*p* = 0.611). Women were found to be misusing sleeping pills more often than men, specifically by taking them in larger dosages. A similar study showed that women tend to misuse sleeping pills by self- prescription more than men (29). The exact reason for this finding is yet to be investigated; however, multiple studies point toward the effects of hormones on the circadian rhythm during the luteal phase of the menstrual cycle, when sleep disturbances are common, especially among those with premenstrual dysphoric disorder (30). Nonetheless, another study suggested that women have better sleep quality than men, which should make them misuse sleeping pills less frequently than men (31). More studies are needed to investigate the gender differences in sleep quality and sleeping pill misuse.

Sleeping pills that are available over the counter (e.g., Panadol Night and melatonin) were found to be more commonly used than sleeping pills that require a doctor’s prescription (e.g., Xanax). Similar findings were reported in a study conducted in Saudi Arabia among physicians during the pandemic, wherein melatonin usage was found to be 75.7% (24). It is important to mention that prescription of certain types of sleeping pills (e.g., zolpidem and zaleplon) is associated with an increased risk of suicidal thoughts and suicide attempts (25); therefore, doctors should be careful when prescribing such medications and prescribe them only in specific cases, when needed.

The study provided evidence of factors associated with the misuse of sleeping pills, such as needing of more than 20 min to fall asleep, being in fourth grade, and using stimulants. In this study, students requiring more than 20 min to sleep showed a strong association with misusing sleeping pills (OR: 9.404; 95% CI, 3.434–25.759; *p* < 0.001). This could be related to poor mental health affecting students’ sleep quality, which means that they end up in need of taking sleeping aids. We also found that sleeping pill use is more common among those who reported poor sleep quality (OR: 2.361; 95% CI, 1.49–3.72), as well as participants who slept for 4–6 h per day (OR: 1.74; 95% CI, 1.12–2.70) (1). Sleeping disorders also increase the occurrence of sedative drug use by 14.9% (2). Fourth-year medical students were found to have a higher rate of misuse (OR: 2.303; 95% CI, 0.627–8.453, *p* = 0.209). Furthermore, a previous cross-sectional study (1) showed that third-year students were more likely to abuse sleeping pills than first- and second-year students (OR: 0.49; 95% CI, 0.30–0.79). This may be because, in preclinical years, students are committed to studying more, due to the stressful curriculum, which has a negative impact on their mental health and can cause sleep disorders (3). According to a study conducted in Ethiopia, stimulant users are more likely to use sedative drugs than non-stimulant users (2). Our research showed that there is a significant association between the misuse of sleeping pills and stimulant use (OR: 3.228; 95% CI, 1.337–7.792, *p* = 0.009). This may be due to the adverse effects of stimulant use on the sleep-wake cycle, which then leads to the misuse and abuse of sleeping pills. As stated by Al-Sayed et al. (1), there is no association between the use of sedatives and stimulants (Table 3).

**TABLE 3:** Binary logistic regression model of associations with sleeping pill misuse.

Variable	OR	95% CI	*P*-value
**Stimulants use**			
Yes	3.228	1.337–7.792	0.009
No	–	–	–
**Falling asleep average, minutes**			
<10	–	–	<0.001
10–20	2.142	0.744–6.166	0.158
>20	9.404	3.434–25.759	<0.001
**Academic year**			
2nd year	–	–	0.028
3rd year	2.614	0.636–10.748	0.183
4th year	2.303	0.627–8.453	0.209
5th year	4.156	1.021–16.913	0.047
6th year	8.665	1.870–40.152	0.006

OR, odds ratio; CI, confidence interval.

This study has several limitations. First, it was limited by its cross-sectional survey design. Second, the convenience sampling technique may limit the generalizability of the results. Third, the lack of questionnaire validation also comprised a limitation. Finally, because this study was conducted in only one university and the questionnaire had a low response rate, the representativeness of the sample is negatively affected and the generalizability of the results is limited. Additionally, the accuracy of respondents’ answers could not be assessed owing to the social stigma attached to misuse, even though confidentiality was ensured. In addition, there is a possibility of cognitive denial or distortion in students’ reporting of their sleeping pill misuse. Future research could further investigate the specific patterns of sleeping pill misuse.

## Data availability statement

The original contributions presented in this study are included in the article/supplementary material, further inquiries can be directed to the corresponding author.

## Ethics statement

The King Abdullah International Medical Research Center provided ethical approval for this study (IRB number: SP21J/025/02). All participants provided their informed consent, after we assured them of their anonymity and data confidentiality. The data were gathered anonymously and recorded in an Excel document on Google Forms. No information that may be used to identify a participant was sought. Written informed consent to participate in this study was provided by the participants’ legal guardian/next of kin.

## Author contributions

MMA and RSA: conceptualization. AA, RSA, RIA, SA, and TB: data collection and analysis and writing—original draft preparation. MMA, MYA, and HA: visualization, supervision, and writing—review and editing. All authors read and approved the final version of the manuscript for publication.
